# Prognostic Value of Quantitative Metabolic Metrics on Baseline Pre-Sunitinib FDG PET/CT in Advanced Renal Cell Carcinoma

**DOI:** 10.1371/journal.pone.0153321

**Published:** 2016-04-28

**Authors:** Ryogo Minamimoto, Amir Barkhodari, Lauren Harshman, Sandy Srinivas, Andrew Quon

**Affiliations:** 1 Department of Radiology, Division of Nuclear Medicine, Stanford University School of Medicine, Stanford, CA, United States of America; 2 Department of Radiology, Molecular Imaging Program, Stanford University, Stanford, CA, United States of America; 3 Department of Internal Medicine, Division of Medical Oncology, Harvard Medical School, Boston, MA, United States of America; 4 Department of Internal Medicine, Division of Oncology, Stanford University School of Medicine, Stanford, CA, United States of America; University of Kentucky College of Medicine, UNITED STATES

## Abstract

**Purpose:**

The objective of this study was to prospectively evaluate various quantitative metrics on FDG PET/CT for monitoring sunitinib therapy and predicting prognosis in patients with metastatic renal cell cancer (mRCC).

**Methods:**

Seventeen patients (mean age: 59.0 ± 11.6) prospectively underwent a baseline FDG PET/CT and interim PET/CT after 2 cycles (12 weeks) of sunitinib therapy. We measured the highest maximum standardized uptake value (SUVmax) of all identified lesions (highest SUVmax), sum of SUVmax with maximum six lesions (sum of SUVmax), total lesion glycolysis (TLG) and metabolic tumor volume (MTV) from baseline PET/CT and interim PET/CT, and the % decrease in highest SUVmax of lesion (%Δ highest SUVmax), the % decrease in sum of SUVmax, the % decrease in TLG (%ΔTLG) and the % decrease in MTV (%ΔMTV) between baseline and interim PET/CT, and the imaging results were validated by clinical follow-up at 12 months after completion of therapy for progression free survival (PFS).

**Results:**

At 12 month follow-up, 6/17 (35.3%) patients achieved PFS, while 11/17 (64.7%) patients were deemed to have progression of disease or recurrence within the previous 12 months. At baseline, PET/CT demonstrated metabolically active cancer in all cases. Using baseline PET/CT alone, all of the quantitative imaging metrics were predictive of PFS. Using interim PET/CT, the %Δ highest SUVmax, %Δ sum of SUVmax, and %ΔTLG were also predictive of PFS. Otherwise, interim PET/CT showed no significant difference between the two survival groups regardless of the quantitative metric utilized including MTV and TLG.

**Conclusions:**

Quantitative metabolic measurements on baseline PET/CT appears to be predictive of PFS at 12 months post-therapy in patients scheduled to undergo sunitinib therapy for mRCC. Change between baseline and interim PET/CT also appeared to have prognostic value but otherwise interim PET/CT after 12 weeks of sunitinib did not appear to be predictive of PFS.

## Introduction

Sunitinib targets multiple signaling pathways, resulting in a dual-action antiproliferative and antiangiogenic effect. It simultaneously inhibits multiple receptor tyrosine kinases including platelet-derived growth factor (PDGF) and vascular endothelial growth factor (VEGF) receptors, which play roles in both tumor cell proliferation and angiogenesis. Since Food and Drug Administration (FDA) approval in January 2006 after demonstrating significant prolongation in progression-free survival (PFS) and a trend to an improvement in overall survival compared to interferon-α, sunitinib is one of the most frequently used first line therapy for advanced renal cell cancer (RCC) [1]. Therefore, continued investigation into utilizing various imaging modalities for defining response and predicting long term outcome after sunitinib therapy is paramount.

Conventional tumor imaging, such as with computed tomography (CT) and magnetic resonance imaging (MRI), relies on changes in size to evaluate response to therapy [2]. In clinical trials, CT imaging has been used to evaluate the response of multi kinase inhibitor (MKI) to RCC lesions, according to the Response Evaluation Criteria in Solid Tumors (RECIST) [3, 4]. However tumor shrinkage is less common in MKI treatment of RCC; therefore, RECIST has limited application in evaluating therapeutic response to RCC lesions, and response assessment of mRCC to sunitinib using ^18^F-fluorodeoxyglucose positron emission tomography (FDG PET/CT) has been reported to be a better alternative in several clinical studies [5–10].

Specifically, our objectives were to evaluate the FDG PET/CT measurement parameters for prediction of prognosis after sunitinib therapy in patients with RCC using histopathologic (post-therapy nephrectomy) or clinical follow-up for validation. To investigate these questions, we executed a single arm prospective trial in patients with newly diagnosed advanced renal cell cancer who were scheduled for sunitinib therapy and utilized an extensive panel of quantitative metrics on baseline and interim FDG PET/CT to evaluate the predictive utility of each of these measurements.

## Materials and Methods

### Patients

This study was approved by the Institutional Review Board (IRB) of Stanford University and fully compliant with the Health Insurance Portability and Accountability Act. Written informed consent was obtained from all patients before participation.

Seventeen previously untreated adult patients with advanced stage IV RCC were prospectively recruited. The demographics of the population were 5 females and 12 males with a mean age 59 ± 12 (range 34–75 years old). Patient inclusion criteria were the following: (a) Pathologic diagnosis of RCC, (b) Advanced (stage IV) RCC, (c) Karnofsky performance status of (KPS>70), (d) Consent to participate in the clinical trial. Exclusion criteria consisted of patients with either (a) uncontrolled hypertension or cardiac disease, (b) and/or history of bleeding diathesis.

Seventeen patients were enrolled and underwent baseline FDG PET/CT prior to initiation of sunitinib therapy. Of the 17 patients, 12 patients then underwent 2 cycles of sunitinib therapy (a total of 12 weeks) and response was evaluated using FDG PET/CT (interim PET/CT). Five patients did not undergo interim PET/CT because of scheduling conflicts and/or concerns regarding radiation exposure from repeated medical imaging. The average injected FDG dose was 14.1 ± 2.2 mCi (range: 10.0–18.0 mCi) for baseline PET/CT and 12.7 ± 2.0 (range: 8.4–14.7 mCi) for interim PET/CT.

During sunitinib therapy, patients with disease progression by size criteria on any images were discontinued from the study and offered second line therapy. FDG PET/CT was analyzed separately from the clinical decision making and correlated to the histopathologic findings for those patients who underwent a nephrectomy or clinical follow-up for those who did not undergo kidney resection.

### Scanning and Image Analysis

The PET/CT acquisition was obtained in 2D mode using a GE Discovery LS 4-detector scanner (GE Healthcare, Waukesha, WI). The CT portion of the exam was not performed with contrast and was used purely for attenuation correction and to aid anatomical localization on the PET images. PET/CT scanning was performed for all 17 patients at baseline within 4 weeks prior to initiation of therapy and for 12 patients after 2 cycles of sunitinib therapy.

On baseline PET/CT, we measured maximum standardized uptake value (SUVmax) of the lesion with the most intense FDG uptake (highest SUVmax), sum of SUVmax of the six highest SUVmax lesions (sum of SUVmax), total lesion glycolysis (TLG) and total metabolic tumor volume (MTV). These assessed lesions were regarded as “targeted lesions” on interim PET/CT.

On the interim PET/CT, we measured highest SUVmax, sum of SUVmax for up to six lesions with the most intense FDG activity, the percentage decrease in SUVmax of targeted lesion between baseline and interim PET/CT (%Δ highest SUVmax), the percentage decrease in sum of SUVmax for the target lesions (%Δ sum of SUVmax), TLG, the percentage decrease in TLG between baseline and interim PET/CT (%ΔTLG), MTV and the percentage decrease in MTV between baseline and interim PET/CT (%ΔMTV). Under these measurements, baseline index or “target” lesions are identified and compared on subsequent PET/CT scans. Reference physiologic SUV values were calculated from an ROI with diameter of 3 cm in normal liver and left atrium for each patient both in baseline and interim PET/CT.

Response on the interim PET/CT was determined semi-quantitatively by measuring changes in tumor intensity as defined by the European Organization for Research and Treatment of Cancer (EORTC) criteria [11]. Under EORTC criteria, baseline index or “target” lesions are identified and compared on subsequent PET/CT scans.

The SUVmax, TLG and MTV of lesions were measured with the PETedge tool MIMvista software (MIM Software Inc., Cleveland, OH) that is a gradient-based tumor segmentation method. Additionally, two board certified Nuclear Medicine physicians with expertise in oncologic PET/CT imaging reviewed all images using visual assessment and gestalt interpretation and produced a consensus impression.

### Clinical Follow-up and Validation

Patients were followed-up for a maximum of 12 months after the initiation of sunitinib therapy. All patients underwent a diagnostic CT or MRI and clinical assessment at 12 months after initiation of sunitinib to evaluate disease status. Clinical assessment included an integrated evaluation of symptoms, laboratory values, and imaging. Patients were scored as (1) improved or stable, or (2) progressed or recurrence at 12 months after initiation of sunitinib.

### Statistical analysis

The study was planned as a pilot feasibility study, and thus no power calculations were made. The target recruitment was over a 2-year period.

The differences between baseline and post-cycle 2 PET/CT were compared using the Mann–Whitney U test. Receiver operating characteristic curve (ROC) analysis was used to obtain suitable cutoff points for highest SUVmax, %Δ highest SUVmax, sum of SUVmax, %Δ sum of SUVmax, MTV, TLG, %ΔMTV　and %ΔTLG. Differences in the Az value among the reference regions for the lesion and between visual assessment and the quantitative value were compared using the test for the equality of ROC areas [12]. The p-values calculated were two-sided, and p < .05 was considered to be indicative of statistical significance.

## Results

The characteristic of enrolled patients are shown in Table 1.

**Table 1:** Patient Demographics and Data.

Index	Value
Age	59.0 ± 11.6
Sex (Male: Female)	12: 5
Baseline PET	17
Interim PET	12
Interval between baseline and interim (days)	92.2 ± 22.6
PFS less than 12 months	11
PFS more than 12 months	6

At baseline, FDG PET/CT demonstrated metabolically active cancer in all cases. The average SUV_max_ of the target lesions in all patients at baseline was 9.8 ± 5.7 (range 2.4–25.3). Reference physiologic SUV values were measured within the liver (average SUVmax of 3.4 ± 0.8 and average SUVmean of 2.3 ± 0.6), and within the left atrium (average SUVmax of 2.4 ± 0.6 and average SUVmean of 1.7 ± 0.4).

At 12 months follow-up, 6/17 (35.3%) patients achieved PFS, while 11/17 (64.7%) patients were deemed to have progression of disease or recurrence within the previous 12 months. Among 12 patients who underwent interim PET/CT, 4/12 (33.3%) patients achieved PFS, while 8/12 (66.7%) patients were deemed to have progression of disease or recurrence within the previous 12 months.

Results are shown on Table 2.

**Table 2:** Comparison of Metrics for Prediction of Progression Free Survival.

Assessment	Index	PFS more than 12 months (n = 6)	PFS less than 12 months (n = 11)	P value
Baseline PET/CT (n = 17)	Highest SUVmax	6.3 ± 3.7	13.1 ± 6.3	0.01
	Sum of SUVmax	11.8 ± 10.2	48.3 ± 35.7	0.02
	TLG (g·10^−3^)	372.3 ± 756.6	2490.1 ± 3257.1	0.03
	MTV (ml)	191.4 ± 16.6	527.6 ± 504.2	0.03
Baseline PET/CT (n = 12)	Highest SUVmax	5.9 ± 4.1	12.2 ± 7.1	0.06
	Sum of SUVmax	10.6 ± 12.3	48.1 ± 42.2	0.06
	TLG (g·10^−3^)	39.1 ± 61.7	2977.5 ± 3760.9	0.02
	MTV (ml)	6.3 ± 8.7	643.6 ± 553.4	0.02
Interim PET/CT	Highest SUVmax	7.1 ± 5.3	12.2 ± 35.4	0.31
	Sum of SUVmax	7.1 ± 8.2	12.2 ± 14.2	0.61
	TLG (g·10^−3^)	37.5 ± 58.9	1300.5 ± 21453.2	0.09
	MTV (ml)	5.4 ± 7.1	463.1 ± 440.2	0.06
Change between baseline and interim PET/CT	%Δ highest SUVmax	-18.9 ± 15.1	34.0 ± 39.7	0.03
	%Δ sum of SUVmax	-16.2 ± 14.5	34.0 ± 39.7	0.02
	%Δ TLG	2.4 ± 2.3	38.1 ± 79.0	0.04
	%Δ MTV	-0.8 ± 12.9	57.0 ± 26.0	0.09

PFS: progression free survival, TLG: total lesion glycolysis, MTV: metabolic tumor volume

All of the quantitative metrics used on baseline PET/CT were predictive of PFS at 12 months and achieved statistical significance. In the 12 patients were we performed both baseline and interim PET/CT, MTV and TLG were predictive of PFS at 12 months and achieved statistical significance. Using interim PET/CT, several quantitative metrics that evaluate changes in metabolic activity (including %Δ highest SUVmax, %Δ sum of SUVmax and %ΔTLG) between baseline and interim PET/CT were also predictive of PFS. However, MTV on interim PET/CT was not predictive of response.

The results based on ROC analysis are shown on Table 3.

**Table 3:** Results Based on ROC Analysis for Predicting Progression Free Survival at 12 Months.

Assessment	Index	Cut off value	Sensitivity	Specificity	PPV	NPV	Accuracy	AUC
Baseline PET/CT	Highest SUVmax	5.3	100.0	66.7	84.6	100.0	88.2	0.83
(n = 17)	Sum of SUVmax	16.4	90.9	83.3	90.9	83.3	88.2	0.87
	TLG (g·10^−3^)	146.0	81.8	83.3	90.0	71.4	82.4	0.83
	MTV (ml)	22.0	81.8	83.3	90.0	71.4	82.4	0.83
Baseline PET/CT	Highest SUVmax	6.0	100.0	75.0	88.9	100.0	91.7	0.88
(n = 12)	Sum of SUVmax	6.0	100.0	75.0	88.9	100.0	91.7	0.88
	TLG (g·10^−3^)	146.0	75.0	100.0	100.0	66.7	83.3	0.88
	MTV (ml)	22.0	75.0	100.0	100.0	66.7	83.3	0.88
Interim PET/CT	Highest SUVmax	3.8	87.5	50.0	77.8	66.7	75.0	0.69
	Sum of SUVmax	7.1	75.0	75.0	85.7	60.0	75.0	0.75
	TLG (g·10^−3^)	321.0	62.5	100.0	100.0	57.1	75.0	0.82
	MTV (ml)	109.0	75.0	100.0	100.0	66.7	83.3	0.88
Change between	%Δ highest SUVmax	0.0	87.5	100.0	100.0	80.0	91.7	0.94
baseline and	%Δ Sum of SUVmax	0.0	87.5	100.0	100.0	80.0	91.7	0.94
interim PET/CT (%)	%Δ TLG	5.0	87.5	100.0	100.0	80.0	91.7	0.94
	%Δ MTV	17.0	75.0	100.0	100.0	66.7	83.3	0.88

ROC: Receiver Operating Characteristic, PFS: progression free survival, PPV: positive predictive value, NPV: negative predictive value, AUC: area under the curve, TLG: total lesion glycolysis, MTV: metabolic tumor volume

**Fig 1:**
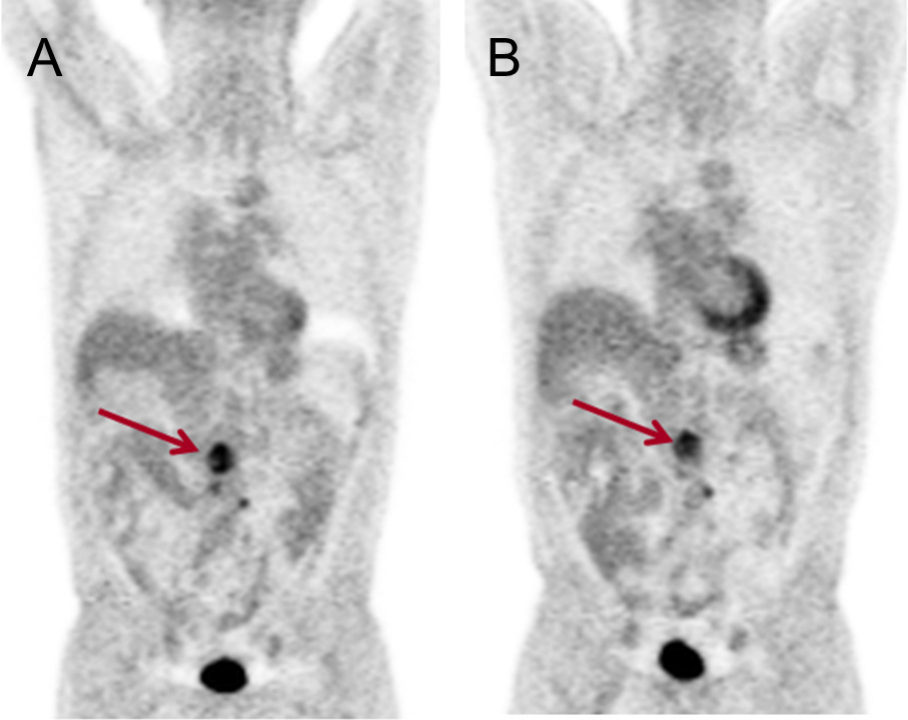
Baseline and post-cycle 2 FDG PET demonstrates improved FDG activity within several abdominal foci at the interim therapy scan (red arrows). Patient experienced PFS at 12 months clinical evaluation. Size measurements on post-cycle 2 MRI (not shown) depicted lesion shrinkage.

**Fig 2:**
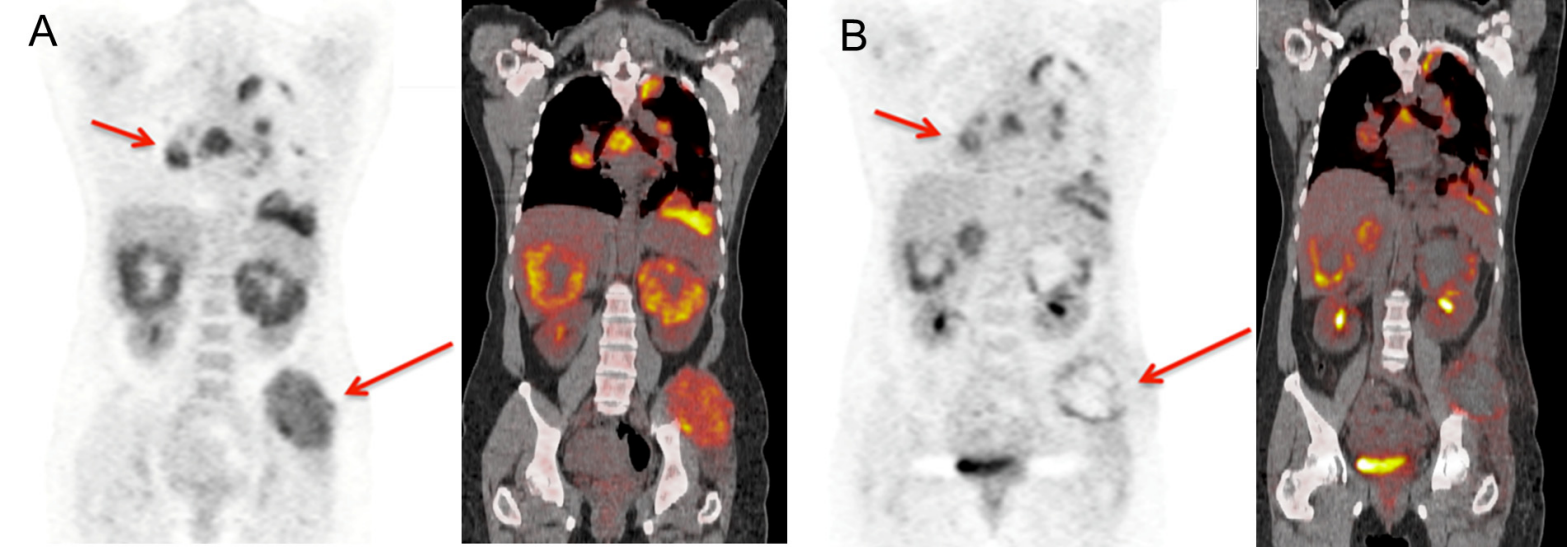
Baseline and post-cycle 2 FDG PET/CT demonstrates improved metabolic activity through multiple bulky foci at the interim therapy scan (red arrows). Size measurements on post-cycle 2 MRI (not shown) had also depicted overall lesion shrinkage. However, patient experienced early progression at 6 months.

On baseline PET/CT, Sum of SUVmax scored the highest area under the curve (AUC), but no other quantitative index showed statistical significance for predicting prognosis (p = 0.69). On interim PET/CT, MTV showed highest AUC. Of the quantitative metrics based on changes between baseline and interim PET/CT, %ΔSUVmax, %Δ Sum of SUVmax, and %ΔTLG showed the highest AUC. Amongst these best performing metrics, no difference could be calculated because of inadequate sample size (p>0.08). The NPV of interim PET/CT for predicting PFS at 12 months (range 57.1–66.7%) was lower than the other indexes. The change between baseline and interim PET/CT showed the highest PPV (100.0%) for predicting disease progression at 12 months (Figs 1 and 2).

Table 4 shows the results based on EORTC criteria for predicting PFS at 12 months.

**Table 4:** Result based on EORTC for predicting progression free survival at 12 months (n = 12).

Interim PET/CT	Sensitivity	Specificity	PPV	NPV	Accuracy	AUC
SD, PR and CR	62.5	100.0	100.0	57.1	75.0	0.81
PR and CR	100.0	50.0	80.0	100.0	83.3	0.75

EORTC: European Organization for Research and Treatment of Cancer, PFS: progression free survival, PPV: positive predictive value, NPV: negative predictive value, AUC: area under the curve, SD: stable disease, PR: partial response, CR: complete response

After cycle 2 of sunitinib, 1/12 patients had complete response (CR), 4/12 patients had partial response (PR), 1/12 patients had progressive disease (PD), and 6/12 patients had stable disease (SD) based on EORTC criteria. Of the patients that had a PR, the mean change of SUVmax in targeted lesions was 6.0 ± 3.3. EORTC criteria present relatively high AUC and accuracy, but lower than other indexes on baseline PET.

## Discussion

The approach to RCC treatment has dramatically changed after the appearance of targeted cancer therapy such as multikinase inhibitors [1, 13–15], and several treatment options such as with mammalian target of rapamycin (mTOR) inhibitor have been developed for advanced RCC [16–19].

The specific aim in this study was to determine whether baseline PET/CT and/or interim PET/CT could predict the prognosis and 12 month outcome of patients with advanced RCC undergoing sunitinib therapy, and what would be the most reliable quantitative parameters or metrics on FDG PET/CT.

In our study, baseline PET/CT alone and changes between baseline and interim PET showed promising results overall. Notably, baseline PET/CT appeared to have the greatest prognostic ability in predicting PFS at 12 months. Potentially, this may indicate interim PET/CT has little clinical value for monitoring sunitinib therapy for advanced RCC, and baseline PET may be regarded as criteria for treatment strategy [20].

Few quantitative indexes on interim PET/CT showed statistically significant ability to predict PFS. Based on the ROC analysis, only MTV and TLG might have prognostic value on interim PET/CT. But even with these metrics, our results still suggest low sensitivity and NPV when using interim PET/CT. Therefore, interim PET/CT alone does not appear to have adequate predictive value. Assessment of the interval change between baseline and interim PET/CT did show promising results as a prognostic index, specifically the change in SUVmax, Sum of SUVmax of the most intense lesions, and change in TLG. When using EORTC criteria (± 25% change of SUVmax for assessment of PD, SD and PR) [11], the AUC was lower than the value estimated by the ROC analysis for baseline and interim PET/CT. This result suggests that revised EORTC response criteria for mRCC may be needed.

We also compared the PET/CT results with dynamic contrast enhanced MRI also performed at baseline and after 2 cycles of sunitinib in this same cohort of patients. However, the MRI was significantly limited by low image quality in lung lesions and lesions near the diaphragm caused by respiratory motion artifact. Our MRI research lab has more recently reported on updated acquisition techniques that better deal with motion artifact and we hope to apply these new protocols to mRCC therapeutic monitoring in future research [21].

Baseline FDG PET/CT appeared to have significant value for predicting patient prognosis [20], in addition to staging and understanding biological character of lesions [22, 23]. Baseline PET/CT, including the advanced quantitative metrics described in this project, appears to have value for treatment selection, managing follow-up time interval, and considering of combination of treatment alternatives.

## Conclusion

Baseline PET/CT and change between baseline and interim PET/CT showed significant prognostic value, including predicting PFS at 12 months after therapy. Interim PET/CT alone did not appear to have significant prognostic value relative to baseline PET/CT, suggesting that it may not be necessary for routine follow-up of sunitinib therapy for advanced RCC. However, larger cohorts are needed in order to confirm these findings.
